# Process optimization of adsorbent production from waste printed circuit board non-metallic fractions based on climate-change impact and cumulative energy demand

**DOI:** 10.1039/d6ra06584h

**Published:** 2026-09-02

**Authors:** Junaid Saleem, Zubair Khalid Baig Moghal, Gordon McKay

**Affiliations:** a Division of Sustainable Development, College of Science and Engineering, Hamad Bin Khalifa University, Qatar Foundation Doha Qatar jsaleem@hbku.edu.qa

## Abstract

The non-metallic fraction (NMF) generated during waste printed circuit board (WPCB) recycling represents a major challenge for electronic-waste management because of its limited recycling opportunities and potential environmental burden. Although NMF can be converted into value-added ion-exchange adsorbents, the environmental implications of adsorbent production remain largely unexplored. To address this knowledge gap, a cradle-to-gate life-cycle assessment was conducted to quantify the climate-change impact (CC) and cumulative energy demand (CED) of ion-exchange adsorbent production from WPCB-derived NMF. Four production routes differing in water consumption and moisture-management strategy were evaluated to identify preferable process configurations and major environmental hotspots. Substantial differences were observed among the evaluated routes. On the basis of 1 kg NMF processed, CC decreased from 33.1 to 5.18 kg CO_2_-eq, while CED decreased from 737 to 126 MJ across the scenarios. Relative to the baseline configuration, the concentrated-paste route reduced CC and CED by approximately 84% and 83%, respectively. Electricity consumption associated with moisture removal and thermochemical conversion was the dominant contributor to both indicators, whereas potassium hydroxide became increasingly important as electricity-related burdens declined. These findings demonstrate that process-design optimization, particularly improved moisture management, can substantially reduce the CC and CED associated with WPCB-derived NMF valorization.

## Abstract 2

The non-metallic fraction (NMF) generated during waste printed circuit board (WPCB) recycling represents a major challenge for electronic-waste management because of its limited recycling opportunities and potential environmental burden.

## Graphical abstract

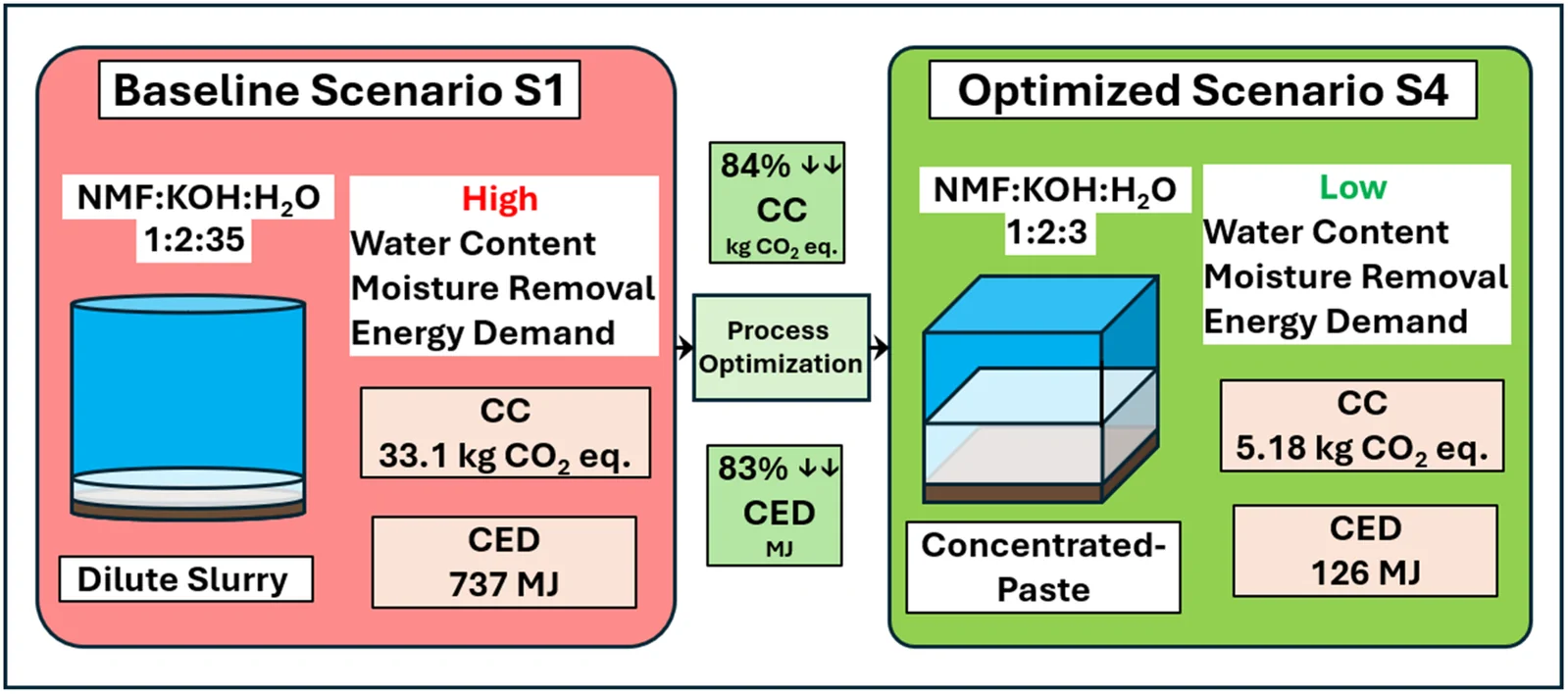

## 1 Introduction

The rapid growth of electrical and electronic equipment (EEE) consumption has resulted in a corresponding increase in waste electrical and electronic equipment (WEEE) generation worldwide.^1,2^ Continuous technological advancement, shortening product lifespans, and increasing consumer demand have accelerated the accumulation of electronic waste, making WEEE one of the fastest-growing waste streams globally. Among the various components of WEEE, waste printed circuit boards (WPCBs) represent one of the most valuable yet environmentally challenging fractions because of their complex composition, heterogeneous structure, and the presence of hazardous substances.^3,4^ Consequently, the development of sustainable WPCB recycling and resource recovery technologies has become an important research priority.

Current WPCB recycling practices primarily focus on recovering valuable metallic constituents through physical, pyrometallurgical, hydrometallurgical, or combined treatment routes. While these approaches can achieve efficient metal recovery, they generate substantial quantities of non-metallic fraction (NMF) as a secondary residue. The NMF typically consists of thermoset resins, glass fibers, fillers, additives, and residual brominated flame retardants.^5–8^ Despite representing a significant proportion of the original WPCB mass, NMF remains considerably underutilized and is frequently disposed of through landfilling or incineration. Such disposal practices not only result in the loss of potentially valuable resources but may also create additional environmental concerns associated with leaching, emissions, and long-term waste management.

To improve resource utilization and support circular-economy objectives, various valorization pathways have been proposed for WPCB-derived NMF. Researchers have investigated the incorporation of NMF into construction materials,^9–12^ geopolymerization^13^ and geopolymer composites,^14,15^ alternative fuels,^16^ carbon materials, and other value-added products.^17^ Among these approaches, the conversion of NMF into functional adsorbent materials has emerged as a particularly attractive strategy because it simultaneously addresses electronic-waste management and water-treatment challenges.^18–23^ By transforming a problematic waste stream into a functional adsorbent, NMF valorization can contribute to both resource recovery and environmental remediation.

Hydroxide-assisted thermochemical conversion has been shown to transform WPCB-derived NMF into porous ion-exchange adsorbents with promising pollutant-removal capabilities.^24^ The resulting materials exhibited favorable physicochemical properties, well-developed porous structures, and high adsorption capacities for heavy metals and other contaminants, demonstrating the technical feasibility of converting WPCB-derived NMF into a high-value functional material.^25^ Subsequent work examined adsorption behavior, activation chemistry, pore development, surface functionalization, brominated-compound degradation, and environmental-emission pathways associated with the conversion process.^26,27^ These investigations significantly improved understanding of the mechanisms governing adsorbent formation and performance. Taken together, the available literature establishes a strong technical foundation for the production and application of NMF-derived adsorbents.

Despite considerable progress in both WPCB recycling and NMF-derived adsorbent development, these research areas have evolved largely independently. Previous environmental assessments have focused on WPCB management systems, recycling pathways, material recovery processes, and manufacturing activities,^28–31^ whereas research on NMF-derived adsorbents has concentrated primarily on material preparation, activation chemistry, surface functionalization, adsorption performance, and pollutant-removal mechanisms. Consequently, although the technical feasibility of producing high-performance NMF-derived ion-exchange adsorbents has been established, the influence of adsorbent production-route design on life-cycle environmental performance remains poorly understood.

This knowledge gap is important because the production of NMF-derived adsorbents involves multiple material- and energy-intensive operations, including chemical activation, drying, thermochemical conversion, washing, and product recovery. Although previous studies successfully optimized adsorbent properties and pollutant-removal performance, environmental optimization has not received comparable attention. To the best of the authors' knowledge, no previous study has systematically evaluated the climate-change impact and cumulative energy demand of NMF-derived ion-exchange adsorbent production or investigated how alternative production-route configurations influence these indicators. As a result, environmentally preferable operating strategies and major environmental hotspots remain unidentified.

Life-cycle assessment (LCA) provides a systematic framework for quantifying environmental impacts across the entire production chain and identifying opportunities for process improvement. In addition to climate-change impact (CC), cumulative energy demand (CED) provides complementary insight into the life-cycle energy requirements of emerging waste-valorization technologies. Unlike direct energy-consumption metrics, CED accounts for both on-site energy use and upstream energy requirements associated with electricity generation, chemical production, water supply, transportation, and wastewater treatment. The combined evaluation of CC and CED provides complementary information on the greenhouse-gas and life-cycle energy implications of alternative production routes, while recognizing that these indicators do not constitute a comprehensive assessment of environmental sustainability.

Accordingly, this study evaluates and optimizes the CC and cumulative-energy performance of adsorbent production from WPCB-derived NMF using LCA. Four alternative production configurations were developed and compared to investigate the influence of process design on environmental performance. CC and CED were selected as the primary assessment indicators, while process-based and input-based contribution analyses were performed to identify environmental hotspots. Sensitivity analysis was further conducted to evaluate the robustness of the scenario ranking within the tested perturbation ranges. The novelty of this work lies in providing the first life-cycle-based evaluation of NMF-derived ion-exchange adsorbent production and the first systematic assessment of how production-route design influences its CC and cumulative-energy performance, thereby shifting the focus from adsorbent-performance optimization to process-level environmental optimization.

## 2 Materials and methods

### 2.1 Adsorbent production route and scenario development

The present study evaluated the CC and CED associated with the production of ion-exchange adsorbents from WPCB-derived NMF. The production route was based on a previously developed potassium hydroxide-assisted thermochemical conversion process for transforming WPCB-derived NMF into functional adsorbent materials for heavy-metal removal.^25,32^ In this route, NMF recovered after corona electrostatic separation was impregnated with potassium hydroxide (KOH) at a KOH : NMF mass ratio of 2 : 1 and mixed with water to form a slurry. The impregnated material was then subjected to thermochemical conversion at 250 °C for 3 h under a nitrogen atmosphere, followed by washing to neutral pH and final drying to obtain the adsorbent product.

The experimentally established thermo-alkaline activation route formed the basis of the baseline configuration (S1), whereas S2–S4 were developed as process-design scenarios to evaluate the effects of moisture management, slurry concentration, and furnace utilization. Accordingly, S2–S4 represent modeled process configurations rather than separately validated production experiments. For comparative modeling, the ANMF yield of 0.901 kg per kg NMF and the functional performance of the resulting adsorbent were assumed to remain unchanged across the four scenarios. Consequently, the results for S2–S4 should be interpreted as prospective environmental-performance estimates associated with process redesign.

Scenario S1 represented the baseline slurry route using an NMF : KOH : water mass ratio of 1 : 2 : 35 without post-impregnation drying. Scenario S2 used the same initial ratio but included partial moisture removal before thermochemical conversion. Scenario S3 also used the same initial ratio but included complete drying before thermochemical conversion. Scenario S4 represented a concentrated-paste route using an NMF : KOH : water mass ratio of 1 : 2 : 3 without post-impregnation drying. These scenarios were designed to identify whether reducing water input and moisture-removal requirements could lower CC and CED.

### 2.2 Goal and scope definition

The objective of this study was to evaluate and optimize the CC and CED performance of WPCB-derived NMF adsorbent production. Particular emphasis was placed on quantifying the effects of water use, slurry concentration, drying requirements, thermochemical conversion, and chemical inputs on these indicators.

The assessment followed a process-based life-cycle assessment approach and included life-cycle inventory development, CC and CED assessment, contribution analysis, sensitivity analysis, and interpretation of results.

This study focused on CC and CED associated with NMF valorization. Other impact categories, such as human toxicity, freshwater ecotoxicity, water consumption, acidification, eutrophication, particulate-matter formation, resource depletion, and hazardous-waste-related impacts, were outside the scope of the present study. Therefore, the results should be interpreted specifically in terms of CC and CED performance rather than as a complete assessment of environmental sustainability.

### 2.3 System boundary and functional unit

A cradle-to-gate system boundary was adopted, covering all processes required to convert WPCB-derived NMF into adsorbent material. The system included NMF transport, pulverization, KOH impregnation, stirring, post-impregnation drying where applicable, thermochemical conversion, nitrogen use, washing, wastewater treatment, and final drying. Upstream production of electricity, KOH, water, nitrogen, and other relevant inputs was included.

Equipment manufacturing, laboratory infrastructure, adsorbent application, regeneration, and end-of-life management were excluded. WPCB-derived NMF was treated as a waste-derived feedstock entering the system with zero upstream environmental burden. Therefore, no allocation was applied to the NMF feedstock.

The functional unit was defined as the valorization of 1 kg of WPCB-derived NMF entering the adsorbent-production process. This feedstock-based functional unit was selected to enable comparison of alternative process configurations on an equivalent NMF-input basis. All foreground material and energy inputs and the corresponding life-cycle impact results were therefore expressed relative to 1 kg of NMF processed.

Under the experimentally established process conditions, 1 kg of NMF produced 0.901 kg of dry activated non-metallic fraction (ANMF). This product yield was retained as an output of the system and was not used to renormalize the inventory. The same NMF input and ANMF yield were applied consistently across all four scenarios, enabling direct comparison of the influence of process configuration on CC and CED.

### 2.4 Life-cycle inventory development

The life-cycle assessment (LCA) was conducted in accordance with ISO 14040 (ref. 33) and ISO 14044.^34^ Background datasets representing electricity generation, chemical production, water supply, nitrogen production, transportation, and wastewater treatment were obtained from the GaBi EN-Professional 2024 database and ecoinvent v3.10.^35^ CC was evaluated using the ReCiPe 2016 Midpoint (H) methodology and expressed in kg CO_2_-eq, while CED was quantified using the energy (net calorific value) indicator and reported in MJ.

The life-cycle inventory was developed using foreground process data derived from the experimentally validated adsorbent-production route together with scenario-specific modifications related to water consumption, moisture-management strategy, and drying requirements. Material inputs included WPCB-derived NMF, KOH, water, and nitrogen gas. Energy inputs consisted primarily of electricity consumption associated with pulverization, stirring, drying, and thermochemical conversion. Additional auxiliary operations included transportation and wastewater treatment.

Scenario-specific inventories were developed for the four production configurations shown in Fig. 1 on the functional-unit basis defined in Section 2.3. The foreground life-cycle inventory and process parameters are presented in Table 1, while the background dataset selections and associated modeling information are provided in the SI (Tables S1–S6).

**Fig. 1:**
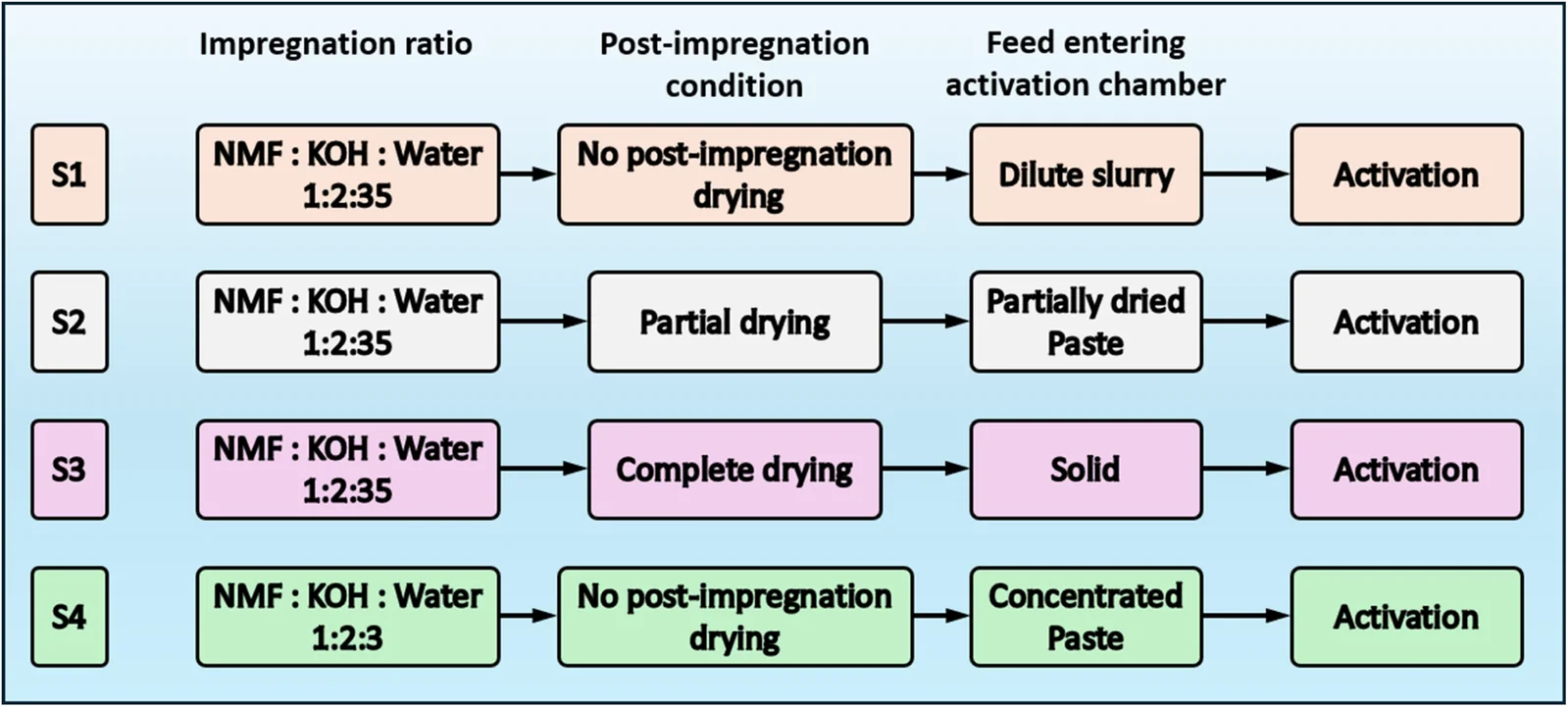
Process configurations evaluated for NMF-derived adsorbent production, showing differences in water consumption, post-impregnation drying, and feed conditions entering thermochemical conversion.

**Table 1:** Foreground inventory and process parameters for the four NMF-derived adsorbent-production scenarios, expressed per functional unit of 1 kg WPCB-derived NMF processed

Inventory item	Unit	S1	S2	S3	S4
WPCB-derived NMF input (functional unit)	kg	1.00	1.00	1.00	1.00
KOH input	kg	2.00	2.00	2.00	2.00
Water for mixing	kg	35.0	35.0	35.0	3.00
Transport of NMF	tkm	0.030	0.030	0.030	0.030
Transport of KOH	tkm	0.060	0.060	0.060	0.060
Pulverization electricity	kWh	0.100	0.100	0.100	0.100
Initial slurry/paste mass	kg	38.0	38.0	38.0	6.00
Stirring electricity	kWh	0.341	0.341	0.341	0.341
Water removed before thermochemical conversion	kg	—	32.0	35.0	—
Water retained before thermochemical conversion	kg	35.0	3.00	—	3.00
Post-impregnation drying electricity	kWh	—	35.20	38.50	—
Feed to thermochemical conversion chamber	kg	38.0	6.00	3.00	6.00
Feed bulk density	kg m^−3^	1000	1000	1000	1000
Chamber fill fraction	—	0.20	0.50	0.60	0.50
Usable chamber volume	m^3^	0.0128	0.0321	0.0385	0.0321
Maximum feed per batch	kg	12.84	32.09	38.51	32.09
Thermochemical feed per kg NMF	kg	38.0	6.00	3.00	6.00
NMF processed per batch	kg	0.338	5.348	12.836	5.348
Furnace electricity per batch	kWh	6.67	6.67	6.67	6.67
Furnace electricity per kg NMF	kWh	19.75	1.25	0.520	1.25
Water evaporated during thermochemical conversion	kg	35.0	3.00	—	3.00
Thermal dehydration electricity	kWh	38.50	3.30	—	3.30
Nitrogen flow rate	L min^−1^	0.300	0.300	0.300	0.300
Total batch time	min	202.5	202.5	202.5	202.5
N_2_ volume per batch	L	60.75	60.75	60.75	60.75
N_2_ mass per batch	kg	0.076	0.076	0.076	0.076
N_2_ input	kg N_2_	0.225	0.014	0.006	0.014
Washing water (20 mL g^−1^ ANMF)	kg	18.02	18.02	18.02	18.02
Wet cake retained water before final drying	kg	0.270	0.270	0.270	0.270
Washing wastewater to treatment	kg	17.75	17.75	17.75	17.75
Water evaporated during final drying	kg	0.270	0.270	0.270	0.270
Final drying electricity	kWh	0.297	0.297	0.297	0.297
**Total electricity input**	**kWh**	**58.99**	**40.49**	**39.76**	**5.29**
**Dry ANMF product**	**kg**	**0.901**	**0.901**	**0.901**	**0.901**

### 2.5 CC and CED assessment

CED represented the cumulative life-cycle energy requirement associated with all material and energy inputs within the system boundary and was distinguished from direct process-electricity consumption. Both CC and CED results were reported on the functional-unit basis of 1 kg WPCB-derived NMF processed and were not renormalized to 1 kg of ANMF produced.

### 2.6 Contribution analysis

Contribution analysis was performed to identify the main drivers of CC and CED. Process-based analysis evaluated the relative contributions of major production stages, whereas input-based analysis evaluated the contributions of key inputs, including electricity, KOH, water, nitrogen, transportation, and wastewater treatment. These analyses were used to identify environmental hotspots and explain differences among the four production scenarios.

### 2.7 Sensitivity analysis

Sensitivity analysis was conducted to evaluate the robustness of the scenario ranking and the influence of key inventory assumptions on environmental performance. Three parameters were varied: electricity consumption (±5%), drying requirements (±10%), and KOH consumption (±10%). The narrower range for electricity reflects the comparatively well-constrained equipment-energy calculations, whereas the wider ranges for drying and KOH reflect greater operational variability associated with moisture content, heat losses, drying efficiency, and chemical dosing.

Other parameters, including product yield, furnace loading, feed bulk density, washing-water demand, electricity-grid composition, and adsorbent functional performance, may exhibit greater uncertainty during process scale-up. These parameters were not varied in the present analysis. Accordingly, the sensitivity analysis should be interpreted as a local assessment of scenario-ranking stability within the selected perturbation ranges rather than as a comprehensive uncertainty analysis.

## 3 Results and discussion

### 3.1 Adsorbent performance and process basis

The production route evaluated in this study was based on a previously optimized thermo-alkaline activation process for converting WPCB-derived NMF into ion-exchange adsorbents. Previous investigations demonstrated that KOH loading strongly influences pore development, mesoporosity, and formation of ion-exchange functional groups, with a KOH : NMF ratio of 2 : 1 identified as an effective condition for producing high-performance adsorbents.^26^ Lower KOH loadings resulted in incomplete activation and reduced ion-exchange-site formation, whereas excessive KOH dosages may promote structural degradation and pore-wall destruction (Fig. 2).

**Fig. 2:**
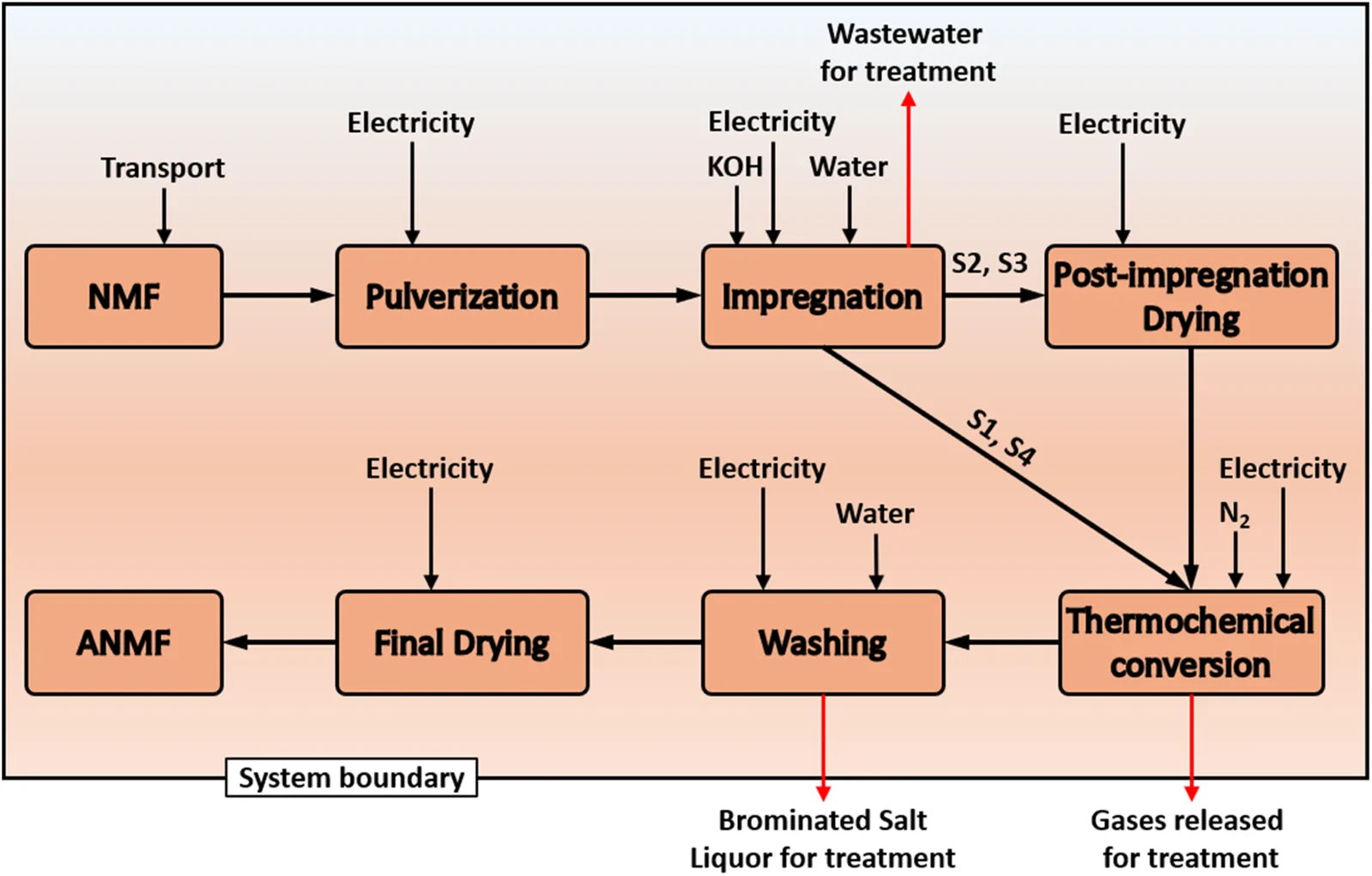
System boundary and process flow diagram for the production of activated non-metallic fraction (ANMF) adsorbent from waste printed circuit board non-metallic fractions.

Under these conditions, activated NMF exhibited a Pb^2+^ adsorption capacity of 683.8 mg g^−1^, substantially exceeding those reported for commercial ion-exchange resins. For comparison, Suqing D401 and Lewatit TP207 achieved Pb^2+^ adsorption capacities of 352.2 and 341.9 mg g^−1^, respectively, approximately one-half of the capacity obtained for activated NMF.^25^ These results demonstrate the importance of KOH dosage in determining adsorbent performance and provide the technical basis for retaining the 2 : 1 KOH : NMF ratio across the evaluated scenarios.

### 3.2 CC of alternative production configurations

The CC associated with the four production configurations are presented in Fig. 3. Substantial differences among the scenarios demonstrate the influence of slurry concentration and moisture-management strategy on CC.

**Fig. 3:**
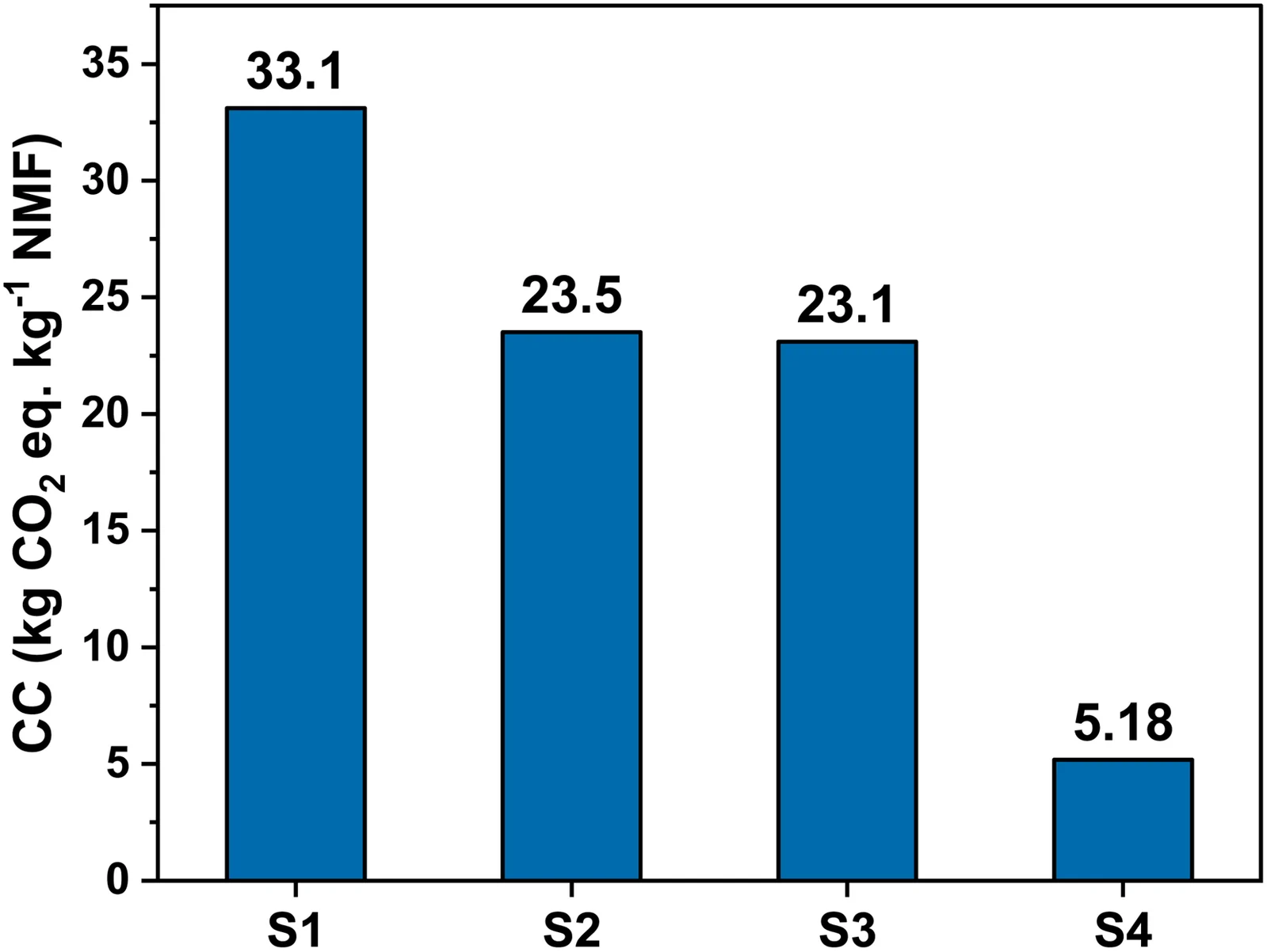
CC associated with the valorization of 1 kg of WPCB-derived NMF under the four evaluated production scenarios.

The baseline configuration (S1) exhibited the highest CC, at 33.1 kg CO_2_-eq kg^−1^ NMF, whereas the concentrated-paste route (S4) achieved the lowest, at 5.18 kg CO_2_-eq kg^−1^ NMF. Intermediate values of 23.5 and 23.1 kg CO_2_-eq kg^−1^ NMF were obtained for S2 and S3, respectively. Relative to S1, S2 and S3 reduced CC by approximately 29% and 30%, respectively, while S4 achieved a reduction of approximately 84%.

The reductions in CC were primarily associated with differences in water input and moisture management. S2 and S3 reduced the moisture entering thermochemical conversion through partial and complete pre-drying, respectively, whereas S4 reduced water addition at the source through the concentrated-paste formulation (NMF : KOH : water = 1 : 2 : 3). The latter strategy produced the greatest reduction in CC, indicating that avoiding excessive water addition was more effective than its subsequent removal. The small difference between S2 (23.5 kg CO_2_-eq kg^−1^ NMF) and S3 (23.1 kg CO_2_-eq kg^−1^ NMF) further indicates that complete pre-drying provided only marginal additional benefit over partial moisture removal.

### 3.3 CED of alternative production configurations

CED for the four production configurations is presented in Fig. 4. Consistent with the CC results, CED decreased from 737 MJ kg^−1^ NMF for S1 to 126 MJ kg^−1^ NMF for S4, with intermediate values of 527 and 518 MJ kg^−1^ NMF for S2 and S3, respectively. Relative to S1, S2 and S3 reduced CED by approximately 28% and 30%, respectively, whereas S4 achieved a reduction of approximately 83%.

**Fig. 4:**
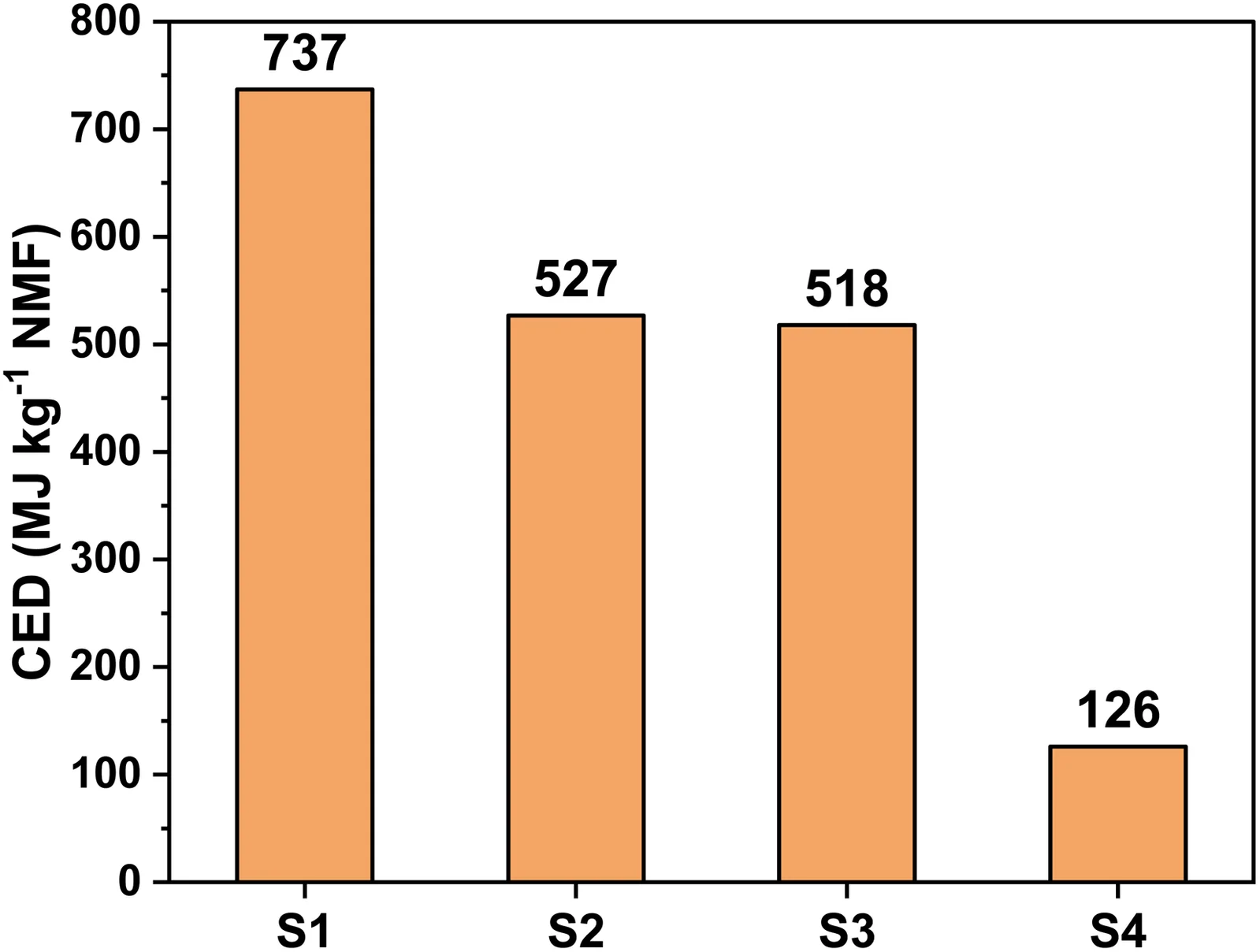
CED associated with the valorization of 1 kg of WPCB-derived NMF under the four evaluated production scenarios.

The close correspondence between the CED and CC trends indicates that the process modifications responsible for reducing life-cycle energy requirements also produced substantial reductions in climate-change impact. The sources of these differences are examined through the contribution analyses in the following sections.

### 3.4 Climate-change hotspots: process-based and input-based contributions

The process-based and input-based contributions to CC are presented in Fig. 5. These analyses identify the production stages and inventory inputs responsible for the differences among the four scenarios.

**Fig. 5:**
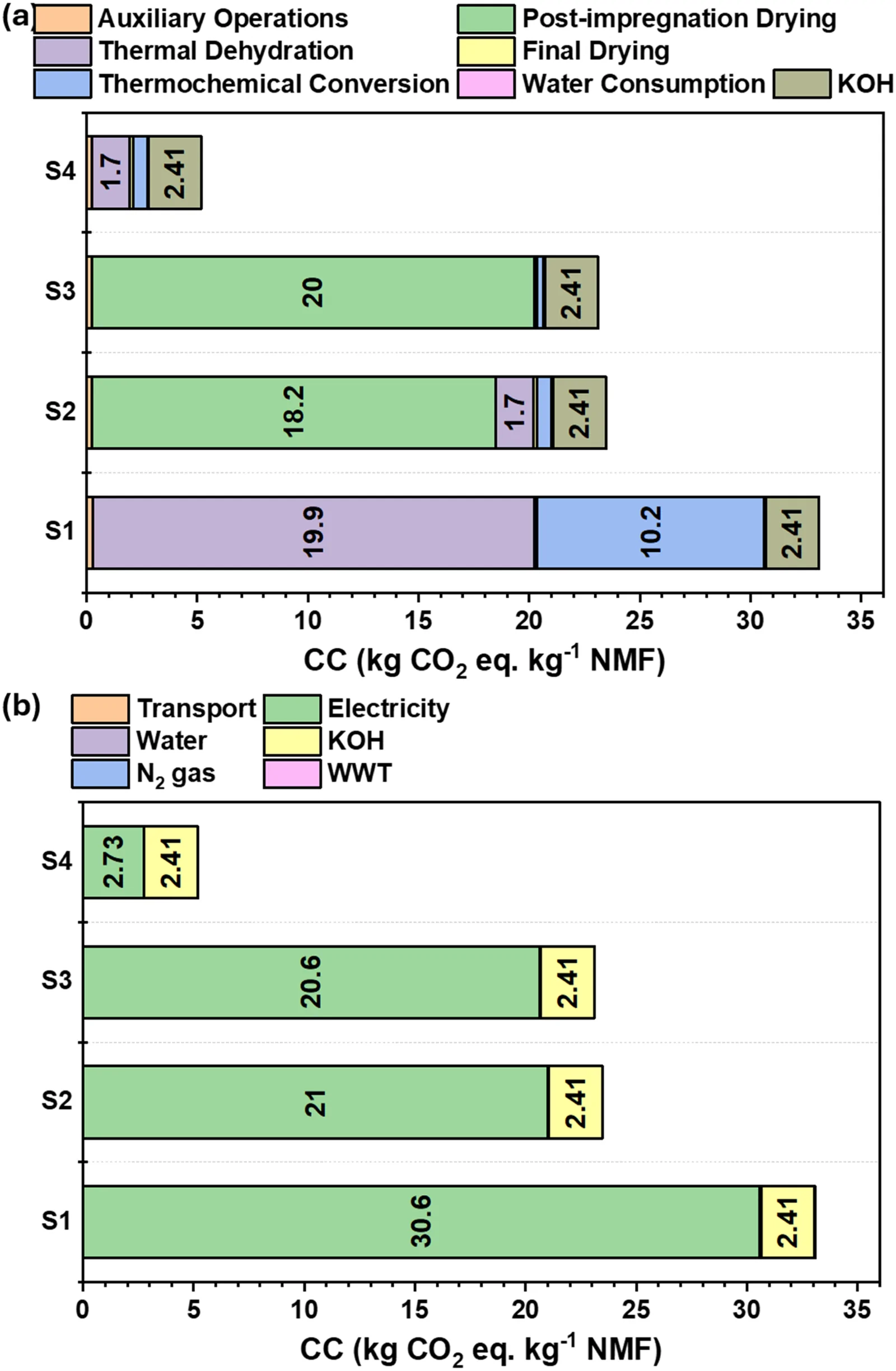
(a) Process-based and (b) input-based contribution analysis of CC for the four evaluated production scenarios.

The process-based contribution analysis (Fig. 5a) showed that thermochemical conversion was the dominant contributor to CC across all scenarios. Its contribution was highest in S1 and decreased substantially in S2 and S3 following moisture removal prior to furnace treatment, with the lowest contribution observed in S4. This trend reflects the progressive reduction in moisture entering thermochemical conversion across the evaluated process configurations. Washing, final drying, and auxiliary operations contributed comparatively small proportions of total CC.

The input-based contribution analysis (Fig. 5b) showed that electricity was the dominant contributor to CC, consistent with the electricity-intensive nature of drying and thermochemical conversion. KOH represented the second-largest contributor and remained constant in absolute terms across the scenarios because the KOH : NMF ratio was maintained at 2 : 1. Contributions from water supply, nitrogen, transportation, and wastewater treatment were comparatively minor.

As electricity-related burdens decreased from S1 to S4, the relative contribution of KOH increased, revealing a shift in the dominant sources of CC. This indicates that chemical consumption becomes increasingly important once the electricity requirements associated with moisture management are reduced. Further reductions in CC may therefore require optimization of KOH consumption or alternative activation strategies while maintaining adsorbent performance.

### 3.5 CED hotspots: process-based and input-based contributions

The process-based and input-based contributions to CED are presented in Fig. 6, providing further insight into the sources of life-cycle energy demand across the four scenarios.

**Fig. 6:**
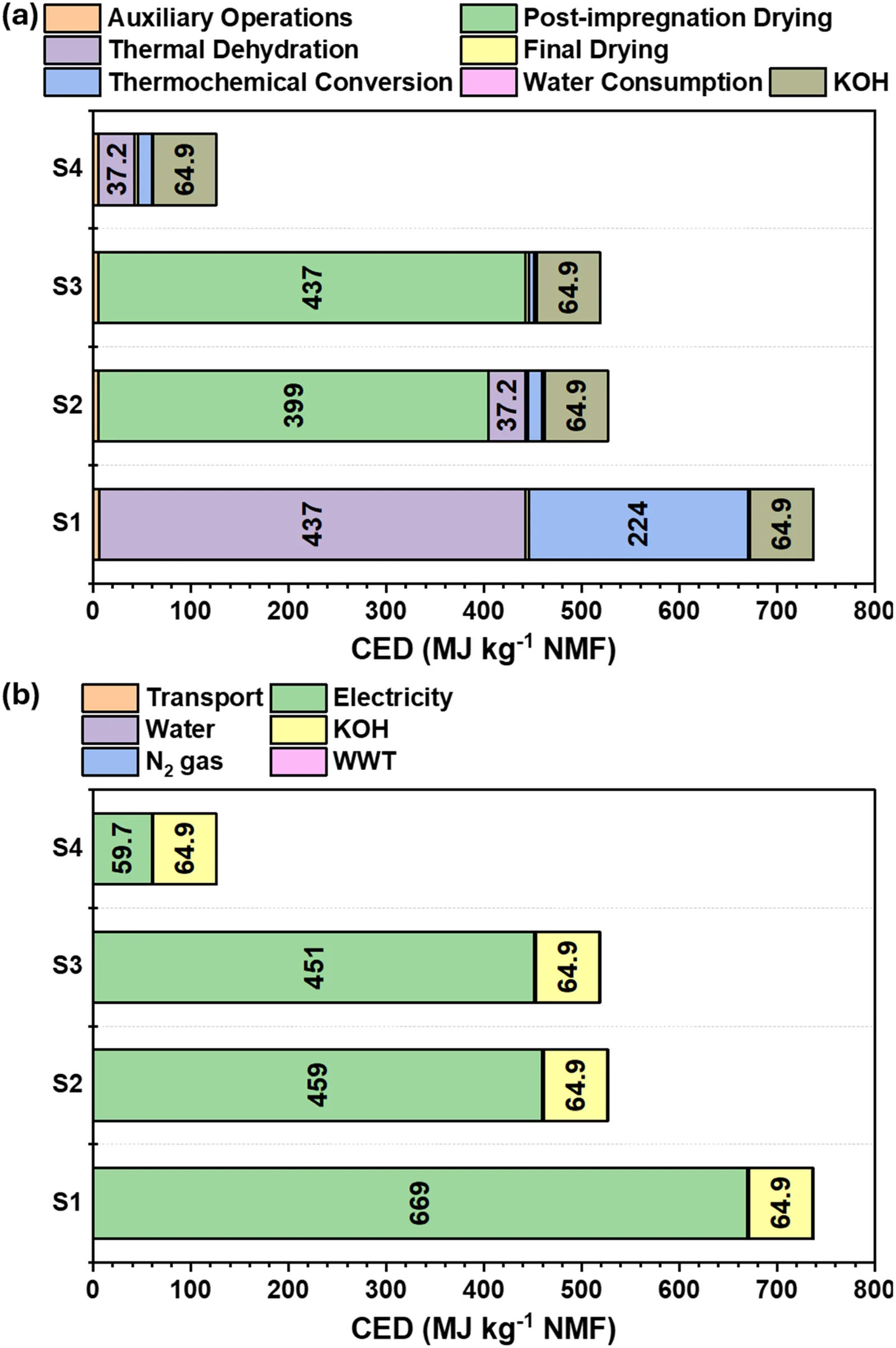
(a) Process-based and (b) input-based contribution analysis of CED for the four evaluated production scenarios.

The process-based contribution analysis (Fig. 6a) showed that thermochemical conversion was the dominant contributor to CED across all scenarios. Its contribution decreased progressively from S1 to S4 as the moisture load entering thermochemical conversion was reduced. Contributions from washing, final drying, and auxiliary operations remained comparatively small across all scenarios.

The input-based contribution analysis (Fig. 6b) identified electricity and KOH as the two principal contributors to CED. Electricity dominated in S1 but decreased substantially as moisture-removal requirements were reduced. In contrast, the absolute contribution of KOH remained constant, while its relative importance increased from S1 to S4.

Notably, the dominant CED hotspot shifted from electricity in S1 to KOH in S4, where KOH became the largest individual contributor. This shift indicates that reducing electricity demand exposes the underlying life-cycle energy burden of chemical production. Further reductions in CED may therefore require optimization of KOH loading or evaluation of alternative activating agents while maintaining adsorbent performance.

### 3.6 Influence of electricity-consuming operations on CC and CED

The contributions of individual electricity-consuming operations to CC and CED are presented in Fig. 7 and 8, respectively. This disaggregation identifies the specific operations underlying the electricity-related hotspots identified in Sections 3.4 and 3.5.

**Fig. 7:**
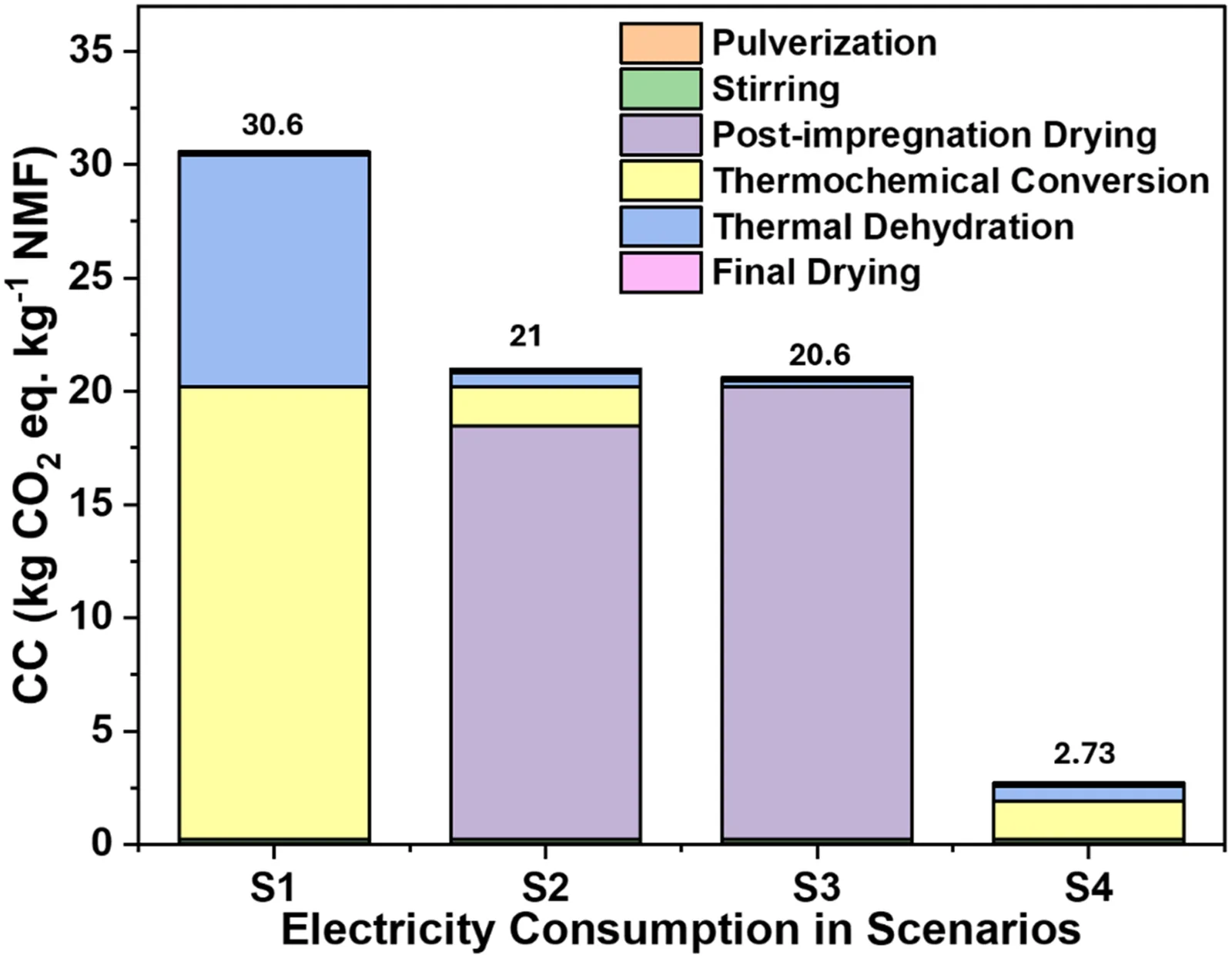
Contribution of individual electricity-consuming operations to CC across the four evaluated scenarios.

**Fig. 8:**
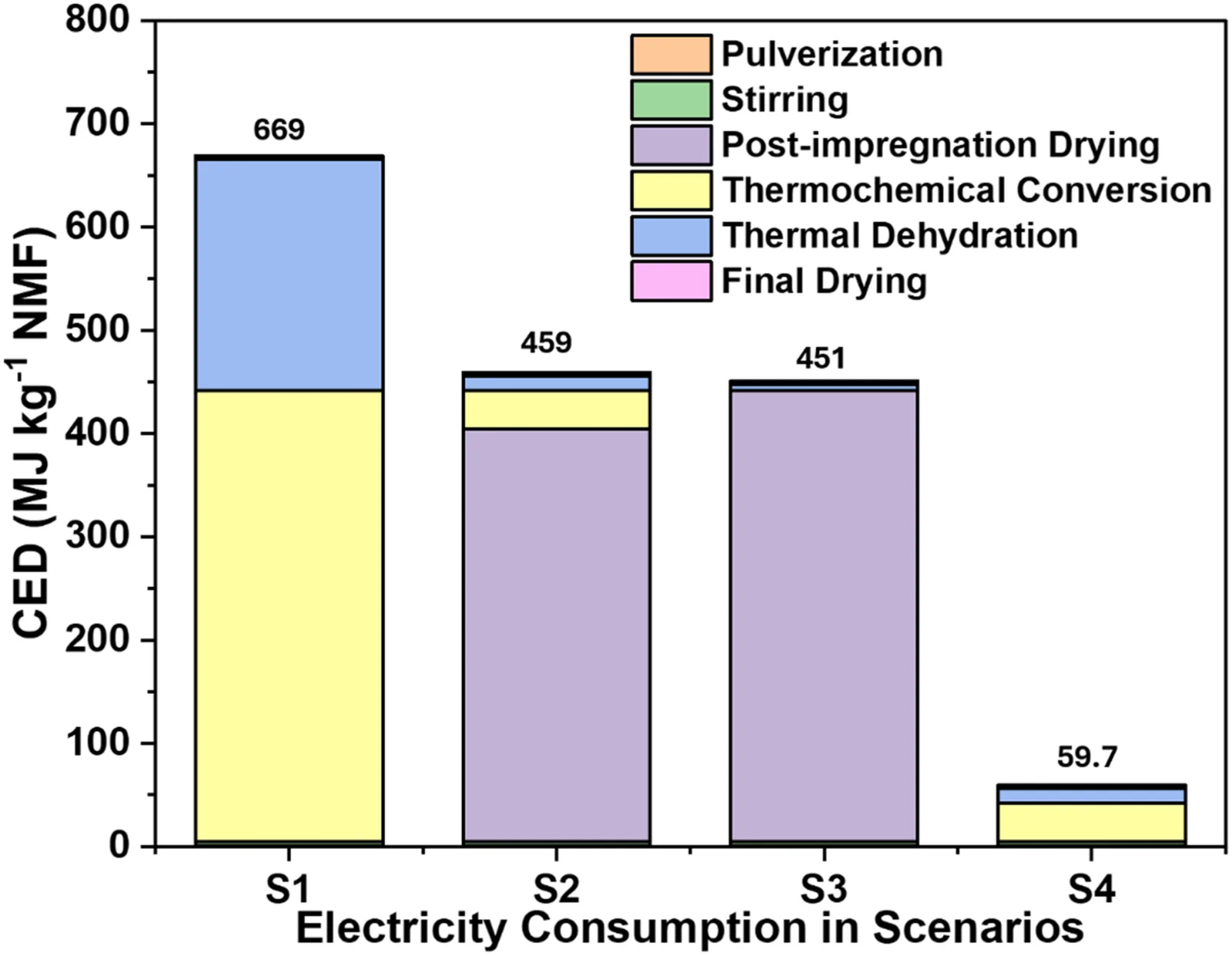
Contribution of individual electricity-consuming operations to CED across the four scenarios.

For both CC and CED, moisture-removal and thermochemical-conversion operations accounted for the majority of electricity-related impacts. In S1, thermal dehydration and thermochemical conversion were the dominant electricity-consuming operations, consistent with the high moisture load of the baseline slurry.

In S2 and S3, pre-drying shifted part of the electricity demand from thermochemical conversion to post-impregnation drying. Nevertheless, the reduction in furnace electricity associated with lower moisture content resulted in lower overall electricity-related CC and CED than in S1.

S4 exhibited the lowest electricity-related contributions because the concentrated-paste configuration substantially reduced the requirements for both moisture removal and thermochemical conversion.

Pulverization, stirring, and final drying made comparatively minor contributions to electricity-related CC and CED and remained relatively stable across the scenarios. Their limited influence indicates that further process-energy optimization should primarily target moisture management and thermochemical conversion.

### 3.7 Sensitivity analysis

The sensitivity analysis results are presented in Fig. 9 for variations in electricity consumption (±5%), drying requirements (±10%), and KOH consumption (±10%).

**Fig. 9:**
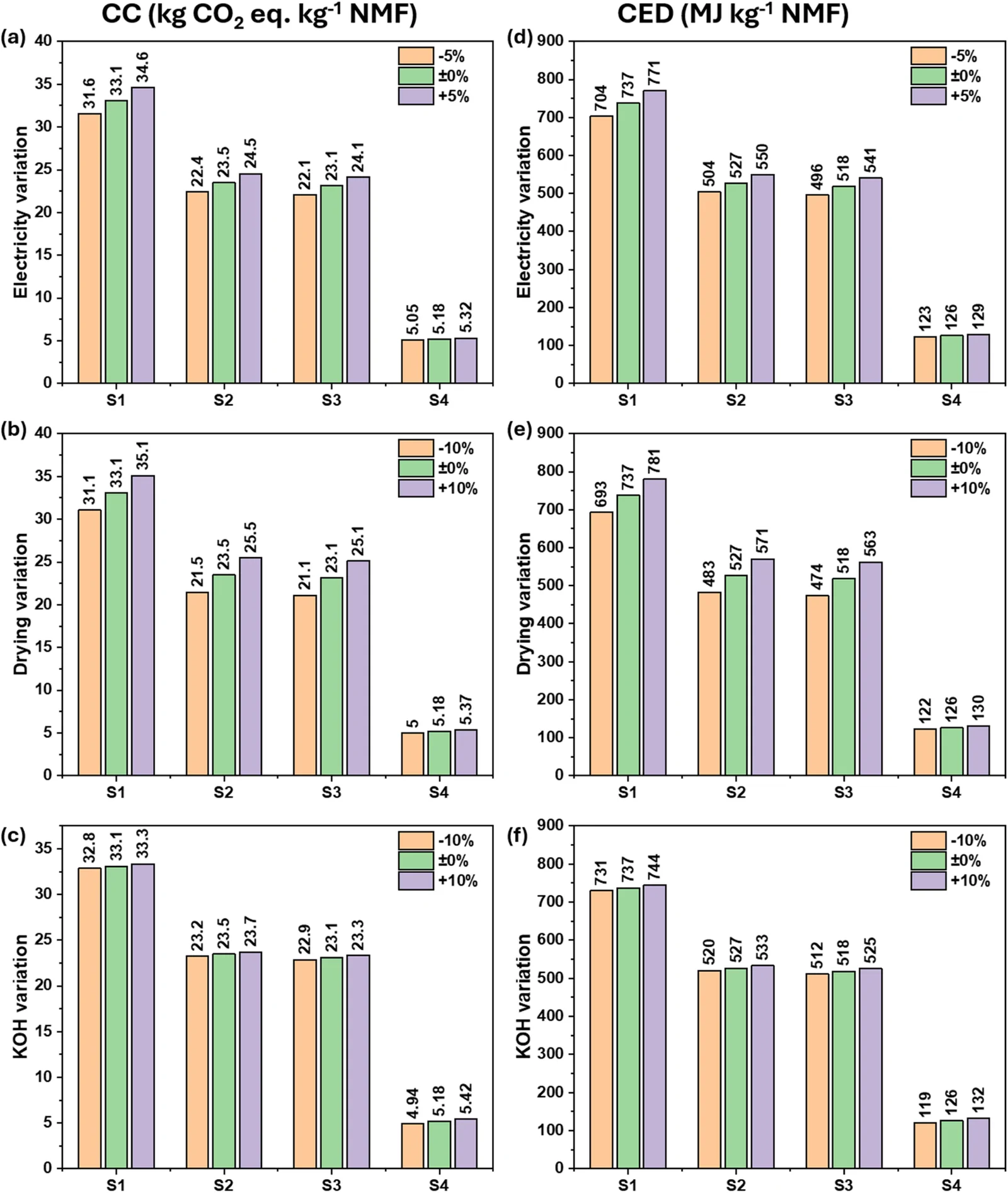
Sensitivity of CC and CED to variations in (a and d) electricity consumption (±5%), (b and e) drying requirements (±10%), and (c and f) KOH consumption (±10%) across the four production scenarios.

The ranking of the four production configurations remained unchanged under all evaluated conditions. S4 consistently exhibited the lowest CC and CED, whereas S1 exhibited the highest, demonstrating that the scenario ranking was stable within the tested perturbation ranges. As noted in Section 2.7, these results represent a local sensitivity assessment rather than a comprehensive uncertainty analysis.

Variations in electricity consumption affected both CC and CED, with larger absolute responses in S1–S3 than in S4. The decreasing sensitivity toward S4 reflects the progressive reduction in electricity requirements across the process configurations.

Variations in drying requirements produced a similar pattern, with greater responses in the higher-moisture scenarios and comparatively limited effects in S4 because of its reduced drying requirements.

Variations in KOH consumption produced comparatively smaller changes in CC but had a more pronounced effect on CED, particularly in scenarios with lower electricity-related burdens. This result further highlights the increasing importance of chemical inputs as process-energy requirements are reduced.

### 3.8 Implications for NMF valorization and process optimization

The results of this study demonstrate that process configuration is a critical determinant of the life-cycle environmental performance of NMF-derived adsorbent production. While previous investigations of WPCB non-metallic fractions have primarily focused on material characterization, adsorption performance, and pollutant-removal efficiency, the present work shows that production-route design can exert an equally important influence on environmental performance. The differences among the evaluated scenarios highlight the value of integrating life-cycle considerations into process design during the early stages of technology development.

Importantly, substantial reductions in CC and CED were achieved without changing the thermochemical conversion conditions or fundamental activation chemistry. Instead, improvements resulted primarily from slurry preparation and moisture management, demonstrating that process-design modifications can substantially reduce life-cycle burdens while retaining the overall production concept.

The shift in relative importance from electricity toward KOH as process-energy requirements decreased illustrates that environmental hotspots are dynamic and may change as a process is optimized. Consequently, life-cycle assessment should be applied iteratively during process development to identify emerging priorities rather than focusing exclusively on the initially dominant burden.

From a broader circular-economy perspective, the results support the potential of WPCB-derived NMF as a secondary resource for producing value-added adsorbent materials. Given that NMF constitutes a substantial fraction of printed circuit board waste and remains underutilized compared with the metallic fraction, improved valorization pathways could enhance resource recovery from WPCB recycling. More broadly, the findings demonstrate how life-cycle assessment can guide process development by identifying opportunities to reduce CC and CED while supporting the valorization of waste-derived materials.

### 3.9 Secondary process streams and emission considerations

Although the present study focused on CC and CED, secondary streams generated during thermochemical conversion and washing are also relevant to the broader environmental performance of ANMF production. These include gaseous emissions, condensed vapors, and washing effluents containing products derived from the transformation of brominated flame retardants and residual organic compounds.^27^

Previous mass-balance studies showed that most potassium introduced through KOH activation remained in the washing liquor, with only a small fraction incorporated into the final adsorbent.^27^ Thermo-alkaline treatment also promoted substantial debromination, converting organic bromine, including that associated with tetrabromobisphenol A, largely into inorganic bromide. Most recovered bromine was concentrated in the washing liquor, with only trace quantities detected in condensed vapors and essentially none remaining in the final adsorbent.^27^ Carbon-containing degradation products, primarily bisphenol A and related compounds, were similarly transferred mainly to the liquid stream, while only minor quantities of light hydrocarbons were detected in gaseous emissions and no significant formation of brominated dioxins or furans was reported under the investigated conditions.^27^ Related WPCB thermochemical studies have also highlighted the potential benefits of bromine recovery.^36^

The concentration of potassium, bromide, and organic degradation products in the washing effluent identifies liquid-stream management as an important consideration for process scale-up. Recovery of alkaline species and bromide, together with wastewater treatment and reuse, could provide opportunities for reducing chemical demand and improving resource recovery. Future life-cycle assessments should incorporate these secondary streams and their treatment or recovery pathways to provide a broader evaluation of NMF-derived adsorbent production.

### 3.10 Future research directions

The hotspot analyses identify chemical consumption as an important target for further process optimization, particularly as electricity-related burdens are reduced. Future research should therefore focus on reducing chemical-related burdens while maintaining adsorbent performance.

The physicochemical properties of NMF-derived materials are strongly influenced by activation conditions and chemical dosage. In particular, the KOH : NMF ratio affects mesoporosity and ion-exchange-site formation, with insufficient activation reported below a ratio of 2 : 1 and potential pore-wall removal at excessive KOH loadings.^26^ Given the increasing contribution of KOH following process-energy optimization, future studies should systematically evaluate lower KOH : NMF ratios, such as 0.5 : 1, 1 : 1, and 1.5 : 1, to determine the trade-off between adsorbent performance and life-cycle burdens.

Alternative thermochemical processing strategies also warrant investigation. Shen *et al.* showed that chemical dosage can influence NMF thermochemical behavior and bromine stabilization, although their study focused on pyrolysis pretreatment rather than adsorbent synthesis.^8^ Future work integrating adsorption performance, bromine management, chemical consumption, and life-cycle assessment could identify process conditions that better balance material functionality with environmental performance.

Finally, future assessments should complement the present feedstock-based functional unit with a performance-based metric, such as environmental impact per unit mass of pollutant removed. This would incorporate differences in adsorption capacity, selectivity, regeneration, and useful lifetime and enable more meaningful comparison with commercial activated carbon, ion-exchange media, and other conventional adsorbents.

## 4 Conclusions

This study evaluated the CC and CED of WPCB-derived NMF valorization through adsorbent production across four alternative process configurations. On the functional-unit basis of 1 kg NMF processed, the baseline slurry route (S1) exhibited the highest burdens, with CC of 33.1 kg CO_2_-eq kg^−1^ NMF and CED of 737 MJ kg^−1^ NMF. In contrast, the concentrated-paste configuration (S4) achieved the lowest values of 5.18 kg CO_2_-eq kg^−1^ NMF and 126 MJ kg^−1^ NMF, corresponding to reductions of approximately 84% and 83%, respectively.

Contribution analyses identified electricity consumption associated with moisture removal and thermochemical conversion as the principal hotspot, while the relative importance of KOH increased as electricity-related burdens were reduced. The results further showed that minimizing water addition at the slurry-preparation stage was more effective than removing excess moisture after its introduction.

Overall, substantial reductions in CC and CED were achieved through process redesign without altering the fundamental activation chemistry. The concentrated-paste configuration therefore provides a promising strategy for improving the climate-change and cumulative-energy performance of NMF-derived adsorbent production. Future optimization should focus on reducing chemical-related burdens while maintaining adsorbent functionality and incorporating secondary-stream management into broader environmental assessments.

## Conflicts of interest

There are no conflicts to declare.

## Supplementary Material

RA-016-D6RA06584H-s001

## Data Availability

The foreground life-cycle inventory, functional-unit definition, process-modeling assumptions, calculation equations, background dataset selections, LCIA settings, and numerical data underlying Fig. 3–9 are provided in the article and supplementary information (SI). Proprietary background life-cycle inventory datasets obtained through licensed databases cannot be redistributed because of licensing restrictions; however, the dataset names, reference flows, and relevant modeling selections are reported to facilitate model reconstruction by appropriately licensed users. Additional calculation information may be obtained from the corresponding author upon reasonable request. Supplementary information is available. See DOI: https://doi.org/10.1039/d6ra06584h.
