# Supplementary material for: Process optimization of adsorbent production from waste printed circuit board non-metallic fractions based on climate-change impact and cumulative energy demand
Source: RSC Adv. 2026 Sep 2;16(43):52578–89. doi: 10.1039/d6ra06584h (PMC13537037; doi:10.1039/d6ra06584h)
Supplement: Supplementary file 1 [file RA-016-D6RA06584H-s001.pdf]

This journal is © The Royal Society of Chemistry 2026

Electronic Supplementary Information

Table S1. Background life-cycle inventory datasets

Foreground input/output	Background dataset/process name	Database	Reference unit	Application
Electricity	Electricity from natural gas	Sphera	MJ	Pulverization, stirring, drying, furnace
KOH	Potassium hydroxide (KOH); electrolysis, from sodium hydroxide and potassium chloride	Sphera	kg	Activation
Water	Water (desalinated; deionised); via ion exchange	Sphera	kg	Mixing/washing
Nitrogen	Nitrogen (gaseous); via cryogenic air separation	Sphera	kg	Furnace purge
Transport	Transport, combination truck, gasoline powered USLCI	Sphera	tkm	30 km transport
Wastewater treatment	Municipal wastewater treatment (mix); mechanical and biological treatment; production mix, at plant; 50% agricultural sludge application, 50% sludge incineration	Sphera	kg	Washing effluent

Table S2. CC and CED scenario results, Figures 3-4

Scenario	CC, kg CO_2-eq kg^{-1} NMF	CED, MJ kg^{-1} NMF
S1	33.1	737
S2	23.5	527
S3	23.1	518
S4	5.18	126

Table S3. Process-based contribution analysis showing CC and CED scenario results. (Figure 5a and 6a)

		Auxiliary Operations	Post-impregnation Drying	Thermal Dehydration	Final Drying	Thermo-chemical Conversion	Water Consumption	KOH
CC (kg CO_2 eq. kg^{-1} NMF)	S1	0.26462	0	19.94953	0.15412	10.24124	0.06913	2.40566
	S2	0.24902	18.24986	1.69967	0.15412	0.64674	0.06913	2.40566
	S3	0.24843	19.96394	0	0.15412	0.26215	0.06913	2.40566
	S4	0.24902	0	1.69967	0.15412	0.64674	0.02741	2.40566
CED (MJ kg^{-1} NMF)	S1	5.86146	0	436.5732	3.3728	224.1181	2.30092	64.85826
	S2	5.33728	399.3778	37.1954	3.3728	14.15317	2.30092	64.85826
	S3	5.3174	436.8884	0	3.3728	5.73692	2.30092	64.85826
	S4	5.33728	0	37.1954	3.3728	14.15317	0.91221	64.85826

Table S4. Inventory-based contribution analysis showing CC and CED scenario results. (Figure 5b and 6b)

		Transport	Electricity	Water	KOH	N_2 gas	WWT
CC (kg CO_2 eq. kg^{-1} NMF)	S1	0.00672	30.57392	0.06913	2.40566	0.01663	0.01224
	S2	0.00672	20.97942	0.06913	2.40566	0.00103	0.01224
	S3	0.00672	20.60924	0.06913	2.40566	4.43E-04	0.01224
	S4	0.00672	2.72956	0.02741	2.40566	0.00103	0.01224
CED (MJ kg^{-1} NMF)	S1	0.09602	669.076	2.30092	64.85826	0.55896	0.19456
	S2	0.09602	459.1111	2.30092	64.85826	0.03478	0.19456
	S3	0.09602	451.01	2.30092	64.85826	0.01491	0.19456
	S4	0.09602	59.7333	0.91221	64.85826	0.03478	0.19456

Table S5. Contribution of individual electricity-consuming operations to climate-change impact (CC) and cumulative energy demand (CED) across the four evaluated scenarios. (Figure 7 and 8).

		Pulverization	Stirring	Post-impregnation Drying	Thermo-chemical Conversion	Thermal Dehydration	Final Drying
CC (kg CO_2 eq. kg^{-1} NMF)	S1	0.05185	0.17717	0	19.94953	10.24124	0.15412
	S2	0.05185	0.17717	18.24986	1.69967	0.64674	0.15412
	S3	0.05185	0.17717	19.96394	0	0.26215	0.15412
	S4	0.05185	0.17717	0	1.69967	0.64674	0.15412
CED (MJ kg^{-1} NMF)	S1	1.13478	3.87715	0	436.5732	224.1181	3.3728
	S2	1.13478	3.87715	399.3778	37.1954	14.15317	3.3728
	S3	1.13478	3.87715	436.8884	0	5.73692	3.3728
	S4	1.13478	3.87715	0	37.1954	14.15317	3.3728

Table S6. Sensitivity analysis with variations in electricity consumption, drying requirements, KOH consumption on climate-change impact (CC) and cumulative energy demand (CED) for the four evaluated production scenarios.

		Electricity variation			Drying variation			KOH variation		
		-5%	±0%	5%	-10%	±0%	10%	-10%	±0%	10%
CC (kg CO_2 eq. kg^{-1} NMF)	S1	31.5556	33.0843	34.613	31.07394	33.0843	35.09467	32.84373	33.0843	33.32487
	S2	22.42524	23.47421	24.52318	21.46384	23.47421	25.48457	23.23364	23.47421	23.71477
	S3	22.07297	23.10343	24.1339	21.09163	23.10343	25.11524	22.86287	23.10343	23.344
	S4	5.04615	5.18262	5.3191	4.99724	5.18262	5.368	4.94206	5.18262	5.42319
CED (MJ kg^{-1} NMF)	S1	703.6309	737.0847	770.5385	693.0901	737.0847	781.0793	730.5989	737.0847	743.5705
	S2	503.6401	526.5956	549.5512	482.601	526.5956	570.5902	520.1098	526.5956	533.0814
	S3	495.9242	518.4747	541.0252	474.4486	518.4747	562.5008	511.9889	518.4747	524.9605
	S4	122.8425	125.8291	128.8158	121.7723	125.8291	129.8859	119.3433	125.8291	132.315
